# Telomere-to-Telomere Genome Assembly of *Tremella sanguinea* Corroborates Its Placement in *Phaeotremella* and Dates Its Divergence (Tremellales)

**DOI:** 10.3390/jof12080602

**Published:** 2026-08-13

**Authors:** Xuelian He, Wenyan Huo, Jianzhao Qi, Lu Dai, Ting Qiao, Liguang Zhang, Yu Liu, Peng Qi, Junzhi Li

**Affiliations:** 1 Fungal Research Center, Shaanxi Provincial Institute of Microbiology, Xi’an 710043, China; xuelhe@163.com (X.H.); huowenyanabace@gmail.com (W.H.); dailutk@126.com (L.D.); syjaqt@163.com (T.Q.); liguang_zhang@163.com (L.Z.); ly137261323@126.com (Y.L.); qipeng_325@163.com (P.Q.); 2 Shaanxi Key Laboratory of Natural Products & Chemical Biology, College of Chemistry & Pharmacy, Northwest A&F University, Yangling 712100, China; qjz@nwafu.edu.cn

**Keywords:** *Tremella sanguinea*, *Phaeotremella*, telomere-to-telomere genome assembly, Tremellales, phylogenomics

## Abstract

*Tremella sanguinea* is a morphologically distinctive gelatinous mycoparasite of *Stereum* within Tremellales whose generic placement has remained unresolved for lack of high-quality genomic resources. We combined Oxford Nanopore long-read sequencing, short-read whole-genome sequencing and Hi-C chromatin conformation capture to assemble the first telomere-to-telomere (T2T) genome of this species: 21.44 Mb in nine gap-free chromosomes, with telomeric repeats recovered at all 18 chromosome ends. Phylogenomic analysis of nine Tremellales genomes, including the type species of *Phaeotremella*, placed *T. sanguinea* within *Phaeotremella* as sister to *P. skinneri* (divergence 24.50 Ma; 95% HPD 22.14–26.89) rather than within *Tremella* sensu stricto, corroborating at genome scale a placement previously indicated by multi-locus data, providing the first divergence-time estimates for the lineage, and reconciling its position with its dark, foliose basidiocarp morphology. Gene family evolution was contraction-biased, most strongly at the *Phaeotremella* crown node (92 expansions vs. 855 contractions). The carbohydrate-active enzyme repertoire of *T. sanguinea* (185 genes) fell within the range reported for Tremellales, whereas families that expanded on its branch were significantly enriched for transport functions, including dipeptide and allantoate transport. These results indicate that *T. sanguinea* acquires host-derived nutrients through uptake of small molecules rather than through an expanded hydrolytic repertoire, and provide genomic evidence for revising its generic placement within the segregate genera of *Tremella* sensu lato.

## 1. Introduction

Tremellales is an order within the class Tremellomycetes of the phylum Basidiomycota, constituting the largest recognized ordinal lineage within this class [1,2]. The order is characterized by gelatinous basidiocarps and a complex life cycle involving alternation between a uninucleate yeast phase and a binucleate hyphal phase [3]. *Tremella fuciformis* and *Naematelia aurantialba* are the most representative and extensively studied edible and medicinal fungi within Tremellales. Both species produce basidiocarps rich in polysaccharides with diverse biological activities including antioxidant, immunomodulatory, and antitumor properties [4,5], and have been developed into commercially cultivated species of considerable economic importance. However, compared with the in-depth research on these representative species, the broader species diversity and genomic resources of Tremellales remain poorly characterized, and the genetic basis and evolutionary history of the great majority of taxa within this order have yet to be systematically elucidated.

Generic delimitation within Tremellales has long been a persistent challenge in fungal systematics. Traditional morphology-based classification relies primarily on basidiocarp morphology, basidiospore characteristics, and hyphal structure; however, the high plasticity of basidiocarp morphology and the difficulty of observing sexual structures under culture conditions have rendered phenotype-based classification frameworks inherently unstable. This inconsistency has become increasingly apparent with the development of molecular systematics. A phylogenetic analysis by Liu et al. based on a seven-gene dataset demonstrated that *Tremella* sensu lato constitutes a polyphyletic assemblage, with morphology-based generic boundaries fundamentally incongruent with true evolutionary relationships, prompting the most comprehensive taxonomic revision in the history of Tremellomycetes—resulting in the recognition of 18 new genera and 7 new families, with 185 taxa recombined [2]. Subsequently, *T. foliacea*, long placed in *Tremella*, was transferred to the newly established genus *Phaeotremella* based on molecular evidence, and multiple cryptic species were further delineated within its morphologically similar species complex [2]. Collectively, these revision cases underscore the fundamental inadequacy of morphological characters alone for resolving phylogenetic affinities within Tremellales, and highlight the indispensable role of molecular and genomic data in establishing a robust and stable classification.

*T. sanguinea* is a distinctive gelatinous fungus within Tremellales, named for its dark reddish-brown to blackish-brown gelatinous foliose basidiocarps that exude amber-red pigments upon rehydration [6]. *T. sanguinea* is an obligate mycoparasite of *Stereum* spp. [7], growing naturally on decaying hardwood logs. Wild resources are scarce and artificial cultivation has yet to overcome the bottleneck of large-scale commercial production [8]. In terms of applied value, the basidiocarps of *T. sanguinea* are rich in diverse bioactive components including polysaccharides, steroids, alkaloids, and phenolic acids [9], and preliminary studies have demonstrated antioxidant and immunomodulatory activities [8,10], indicating considerable potential for further development and utilization. However, *T. sanguinea* has traditionally been placed in *Tremella* based on morphological characters, despite its dark gelatinous basidiocarps and blood-red pigmentation being markedly distinct from those of the type species of *Tremella*. Notably, dark, foliose basidiocarps are characteristic of *Phaeotremella*, the genus segregated from *Tremella* sensu lato for the *T. foliacea* clade [2]; members of this group are typified by dusky, leaf-like fruit bodies [11], a morphology matched by *T. sanguinea*. Coupled with the very limited phylogenetic studies available for this species, its true evolutionary position within Tremellales has long remained unresolved. The absence of high-quality genomic data represents the key bottleneck constraining a deeper resolution of this problem.

In recent years, the combined application of Nanopore long-read sequencing and Hi-C chromatin conformation capture technology has driven a transition in fungal genome assembly quality from draft-level to complete telomere-to-telomere (T2T) assemblies [12]. T2T genomes not only enable complete annotation of coding regions, but also allow accurate resolution of highly repetitive regions including centromeres, telomeres, and subtelomeric regions, providing the highest-quality sequence foundation for studies of chromosomal evolution, comparative genomics, and species diversification [13,14].

Within Tremellales, existing genomic research has focused primarily on *T. fuciformis* and *Naematelia* spp. Genomic resources for *T. fuciformis* have progressively advanced from an early Illumina draft assembly (22.4 Mb, 1063 contigs) [15] to a PacBio HiFi + Hi-C chromosome-level assembly (23.64 Mb, 10 chromosomes) [16] and subsequently a T2T-level diploid assembly [12], with population-level sequencing further revealing the extensive polymorphism and evolutionary origins of accessory chromosomes [17]. For *Naematelia*, a chromosome-level genome assembly of *N. sinensis* (12 chromosomes) was recently reported [18]. Nevertheless, all of these studies have focused on *Tremella* and *Naematelia*; no chromosome-complete genome has been available for *Phaeotremella*, the genus within which *T. sanguinea* is placed here, limiting comparative analysis within this clade. A multi-locus analysis by Chen et al. [7] has already indicated that this species falls within *Phaeotremella* rather than *Tremella* sensu stricto; that inference, however, rested on a small number of loci, was not tested at genome scale, and provided no estimate of divergence time.

To address this gap, the present study employed an integrative strategy combining long-read sequencing, short-read whole-genome sequencing, and Hi-C chromatin conformation capture to generate the first T2T-level genome assembly of *T. sanguinea*, yielding 9 complete gap-free chromosomes with telomeric sequences successfully identified and extended at all 18 chromosome ends, achieving 100% telomere coverage. Building on this assembly, we reconstructed the phylogenetic position of *T. sanguinea* using a framework of single-copy orthologous genes from multiple species, incorporating both maximum likelihood and coalescent-based phylogenetic analyses alongside divergence time estimation. We further conducted comparative genomic analyses encompassing gene family evolutionary dynamics, carbohydrate-active enzyme (CAZyme) composition, and GO functional annotation. Collectively, this work provides the first chromosome-complete genome for a member of *Phaeotremella* and supplies direct genomic evidence for reassessing the generic placement of *T. sanguinea*—a species described on morphological grounds in 1990 and since retained in *Tremella*—within the revised generic framework of Tremellales.

## 2. Materials and Methods

### 2.1. Sample Preparation and Collection

**Figure 1:**
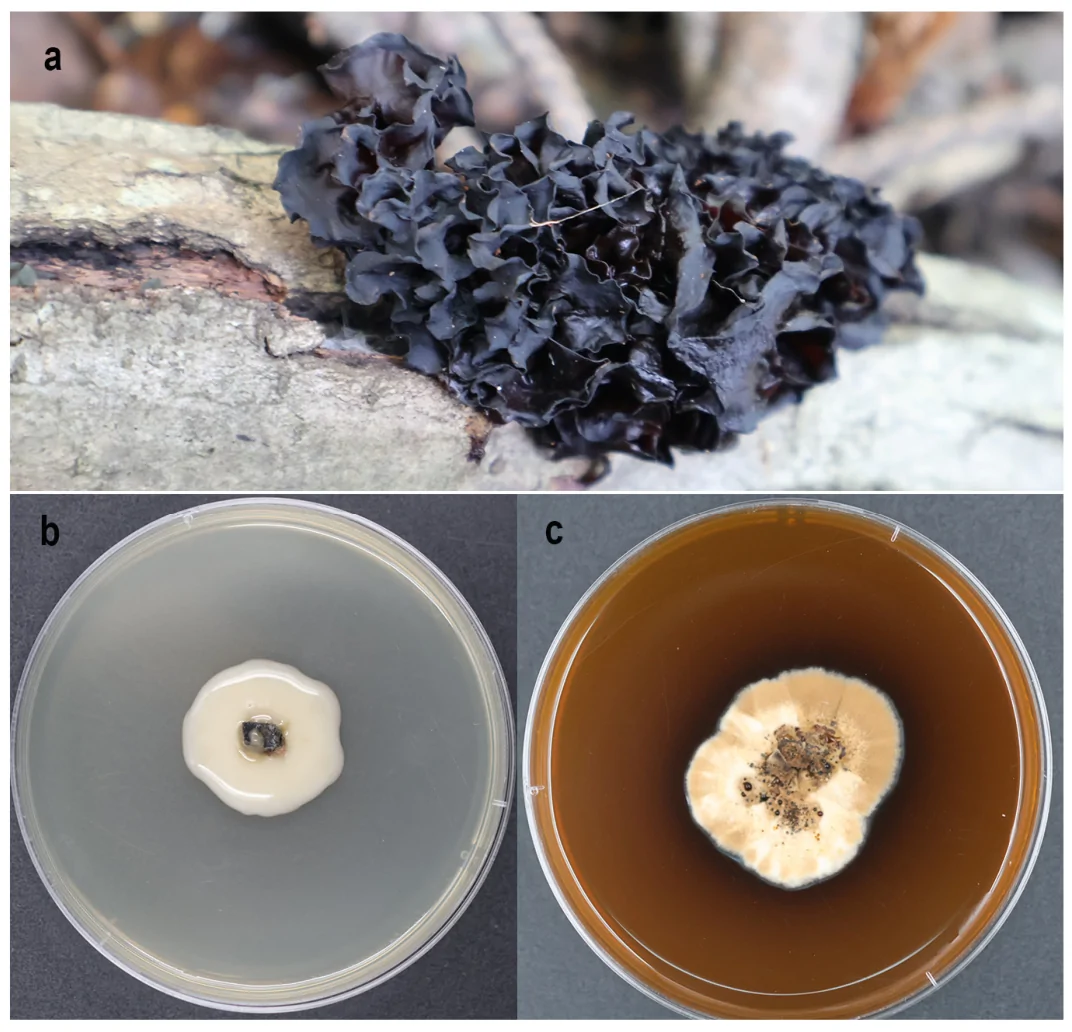
Morphological characteristics and isolation culture of *T. sanguinea*. (**a**) Fresh fruiting bodies collected from the field; (**b**) Monosporic blastoconidial (yeast-phase) culture; (**c**) Dikaryotic mycelium obtained by pairing two compatible monosporic isolates, used only for transcriptome sequencing.

Fresh fruiting bodies of *T. sanguinea* were collected from the Nanniwan region of Yan’an, Shaanxi Province, China (Figure 1). Basidiospores were discharged from fresh fruiting bodies, and a single basidiospore was isolated and purified through three successive rounds of single-colony purification, yielding the monosporic strain YAXE-01 (deposited as SXIM-1424). Species identity was confirmed by morphological characterization combined with internal transcribed spacer (ITS) sequence analysis using primers ITS4/ITS5 (GenBank accession: PV1770701). As is characteristic of *Tremella* and allied genera, *T. sanguinea* proliferates readily in a yeast-like state as blastoconidia (budding cells); blastoconidia germinate into hyphae only rarely, and shaken liquid cultures inoculated with blastoconidia remain entirely in the yeast phase. The monosporic culture is therefore a clonal, monokaryotic (single-nucleus) population of blastoconidia derived from one meiotic product. Additional monosporic isolates were established from the same basidiocarp by the same single-spore procedure and retained for mating tests.

Dikaryotic mycelium was obtained separately, because monosporic cultures proliferate almost exclusively as blastoconidia and yield too little hyphal biomass for RNA extraction. Two compatible monosporic isolates were paired on potato dextrose agar, and the clamp-bearing dikaryotic mycelium that formed at the contact zone was subcultured and used only for transcriptome sequencing. Because blastoconidia continue to bud from hyphal cultures, mycelium-dominated biomass was enriched by allowing the culture to stand so that the mycelial aggregates settled while the blastoconidia remained in suspension; the settled mycelial fraction was then collected and the supernatant discarded. Both culture types were inoculated into potato dextrose (PD) broth and grown at 23 °C with constant shaking for 6 days. Biomass of both types was harvested by centrifugation under aseptic conditions and washed three times with sterile distilled water to remove residual medium components. Genomic DNA for whole-genome sequencing (Nanopore, MGI short-read and Hi-C) was extracted exclusively from the monosporic, monokaryotic blastoconidial culture. For transcriptome sequencing, RNA was extracted from both the monosporic blastoconidial culture and the dikaryotic mycelium (four biological replicates each), so that transcripts expressed in both the yeast and the hyphal state were captured for annotation. All harvested samples were immediately snap-frozen in liquid nitrogen and stored at −80 °C until nucleic acid extraction.

### 2.2. Nucleic Acid Extraction and Quality Assessment

Genomic DNA and total RNA were extracted by Wuhan Benagen Technology Co., Ltd. (Wuhan, China). High-molecular-weight genomic DNA was extracted from the frozen monosporic blastoconidial culture with an in-house SDS-based protocol optimised for fungal ultra-long DNA (no commercial kit): approximately 0.5 g of biomass was ground in liquid nitrogen for 15–20 min, resuspended in tissue lysis buffer, filtered through a 40-µm membrane, and lysed in SDS extraction buffer containing RNase A at 50 °C for 1.5 h; DNA was purified by phenol:chloroform:isoamyl alcohol (25:24:1) followed by chloroform:isoamyl alcohol (24:1), precipitated with 0.8 volume of isopropanol, washed with 75% ethanol and dissolved in elution buffer. The same DNA preparation was used for both long-read (ONT) and short-read (MGI) sequencing. Total RNA was extracted from the frozen blastoconidial and mycelial samples with the E.Z.N.A.^®^ Plant RNA Kit (cat. R6827, Omega Bio-tek, Norcross, GA, USA) according to the manufacturer’s instructions (the kit is validated in-house for fungal material). Nucleic-acid purity and quantity were assessed with a NanoDrop One spectrophotometer and a Qubit 3.0 fluorometer (Thermo Fisher Scientific, Waltham, MA, USA), and integrity was evaluated by agarose gel electrophoresis.

### 2.3. Library Construction and Sequencing

All sequencing libraries were constructed and sequenced by Wuhan Benagen Technology Co., Ltd. For long-read sequencing, ONT libraries were prepared from high-molecular-weight genomic DNA with the amplification-free Ligation Sequencing Barcoding Kit (SQK-NBD114.96; Oxford Nanopore Technologies, Oxford, UK), loaded onto R10.4.1 flow cells and sequenced on a PromethION platform(Oxford Nanopore Technologies, Oxford, UK); base calling used Dorado v0.9.0. For short-read whole-genome sequencing, libraries were prepared with the Hieff NGS OnePot Pro DNA Library Prep Kit V4 (one-step enzymatic fragmentation; Yeasen, Shanghai, China, cat. 12972) and sequenced in PE150 mode on a DNBSEQ-T7 platform (MGI, Shenzhen, China). For chromosome-scale scaffolding, Hi-C libraries were constructed from formaldehyde-crosslinked blastoconidial cells with an in-house restriction-enzyme protocol (no commercial kit): chromatin was digested with DpnII, 5′ overhangs were filled in with biotinylated nucleotides, and spatially proximal fragments were joined by in situ proximity ligation; after crosslink reversal and purification, the DNA was sheared and size-selected to 300–700 bp, and biotinylated junctions were captured on streptavidin magnetic beads. Libraries were quantified (Qubit 3.0, Thermo Fisher Scientific, Waltham, MA, USA; Agilent 2100 Bioanalyzer, Santa Clara, CA, USA and qPCR) and sequenced in PE150 mode on DNBSEQ-T7. For transcriptome sequencing (blastoconidial and mycelial samples, four biological replicates each), libraries were prepared with the VAHTS Universal V10 RNA-seq Library Prep Kit (Premixed Version, V24.1; Vazyme, Nanjing, China, cat. NRM616; non-stranded, oligo-dT mRNA enrichment) and sequenced in PE150 mode on DNBSEQ-T7.

### 2.4. Genome Survey Analysis

Prior to genome assembly, k-mer frequency distribution analysis was performed to characterize the fundamental genomic properties of *T. sanguinea*. K-mer frequency spectra were constructed from quality-filtered short reads using Jellyfish (v2.2.10) [19] with a k-mer size of 21. The resulting frequency histograms were subsequently modeled using GenomeScope 2.0 [20] to estimate genome size, heterozygosity, and repeat content, providing essential parameters for the design of the downstream assembly pipeline. Because the sequenced material is a clonal blastoconidial population derived from a single basidiospore—that is, an asexually amplified population of one meiotic product—the genome is expected to be haploid and homozygous by descent.

### 2.5. Data Quality Control and Genome Assembly

All raw sequencing datasets were subjected to stringent quality control prior to assembly. Short reads from the MGI DNBSEQ platform were processed with fastp (v0.23.2) [21] for adapter trimming and low-quality sequence removal. Hi-C data underwent quality control using Trim Galore (v0.6.7) [22] screened with Filtlong (v0.2.1) [23] to eliminate poor-quality and excessively short fragments.

De novo genome assembly was initiated using NECAT (v0.0.1) [24] for error correction of raw ONT reads and construction of preliminary contigs. The initial contigs were subsequently refined through three iterative rounds of polishing with Racon (v1.4.3) [25], with ONT reads being realigned to the evolving assembly reference using minimap2 (v2.31-0) [26] at each iteration. For single-nucleotide error correction, short reads were aligned to contigs using BWA-MEM [27], followed by sorting and indexing with SAMtools (v1.22.1) [28]. Two additional rounds of base-level correction were then performed using Pilon (v1.24) [29].

Chromosome-scale genome scaffolding was achieved through Hi-C technology. Hi-C alignment data were processed using Juicer (v1.6) [30], followed by scaffolding assembly with 3D-DNA [31]. Assembly quality was evaluated and optimized through manual inspection of Hi-C contact heatmaps in Juicebox Assembly Tools [32], during which misjoins were identified and corrected while redundant contigs were eliminated. The manually curated assembly was converted to chromosome-level FASTA format using the assembly2agp and agp2fasta modules of CPhasing (v0.2.1, https://github.com/wangyibin/CPhasing, accessed on 30 December 2025).

Finally, FungalTeloExtender (v1.0.3, https://github.com/huowenyanabace/FungalTeloExtender, accessed on 23 March 2026) was employed to identify telomeric repeat motifs (AGGGGGTT/AACCCCCT) and append telomeric sequences to chromosome termini, achieving a complete Telomere-to-Telomere (T2T) assembly of the *T. sanguinea* genome.

### 2.6. Genome Assembly Quality Assessment

A comprehensive multi-dimensional evaluation framework was applied to validate the final genome assembly. Gene space completeness was evaluated using BUSCO (v5.4.7) [33] with the fungi_odb10 reference database at both genome and protein levels. Assembly contiguity was assessed by QUAST (v5.0.2) [34] to calculate key metrics including contig number and N50 values. Base-level accuracy was quantified using Merqury (v1.4.1) to compute consensus quality values (consensus QV) as a measure of assembly precision.

Chromosomal structural accuracy was validated using genome-wide Hi-C sequencing data to construct interaction heatmaps. Visualization and verification were performed through Juicer Tools [30] and HiCExplorer (v3.6) [35] to confirm the correctness of inter- and intra-chromosomal three-dimensional organization. Telomere integrity was verified through computational prediction using FungalTeloExtender (v1.0.3, https://github.com/huowenyanabace/FungalTeloExtender, accessed on 23 March 2026) combined with manual inspection, confirming that all chromosome termini contained recognizable telomeric repeat sequences and validating the completeness of the T2T assembly.

For cross-species comparison of gene set completeness across the nine genomes (Table S2), a separate BUSCO analysis was performed in protein mode using the fungi_odb12 database. This updated BUSCO dataset provides broad, balanced taxonomic sampling across fungi and was selected to enable consistent comparative assessment across the nine Tremellales species. For the *T. sanguinea* genome, the fungi_odb12 protein-mode completeness was 95.5%, whereas the fungi_odb10 genome-mode completeness (98.0%) was retained as the primary quality metric for the assembled genome.

### 2.7. Transposable Elements and Repetitive Sequence Annotation

A combined strategy of de novo prediction and homology-based annotation was employed to systematically annotate repetitive sequences across the *T. sanguinea* genome. An RMBLAST database was constructed based on the T2T assembly, and RepeatModeler (v2.0.1) [36] was utilized to build a species-specific repeat library for *T. sanguinea* through iterative search and sequence optimization. This custom library was integrated with known repeat sequences from Repbase (release 20181026) [37], and RepeatMasker (v4.2.2) [38] was subsequently applied in sensitive mode for precise identification and soft-masking of repetitive regions genome-wide.

Annotated repetitive sequences were classified and enumerated by type, including major categories such as retrotransposons (LTR and non-LTR retrotransposons), DNA transposons, rolling circle replication elements, simple sequence repeats (SSRs), low-complexity sequences, and small RNA-associated repeats. The composition, density, chromosomal distribution, and structural patterns of repetitive elements provided essential foundational information for subsequent gene structure prediction, functional annotation, and genomic evolutionary analyses.

### 2.8. Gene Prediction and Functional Annotation

Structural gene prediction for the *T. sanguinea* genome was performed using an integrated strategy combining ab initio prediction with transcriptomic evidence. RNA-seq data were mapped to the genome using HISAT2 (v2.2.1) [39], and the resulting alignments served as empirical evidence for initial gene prediction with BRAKER (v2.1.6) [40]. Final gene models were constructed using MAKER (v3.1.3) [41] by integrating multiple lines of evidence: (1) de novo transcripts assembled from target species RNA-seq data using rnaSPAdes (SPAdes v3.15.5) [42]; (2) homologous protein sequences retrieved from NCBI databases; and (3) Augustus [43] parameters trained and optimized during the BRAKER step.

Functional gene annotation was accomplished using eggNOG-mapper (v2.1.12) [44] based on homologous sequence searches to assign Gene Ontology (GO), Kyoto Encyclopedia of Genes and Genomes (KEGG), and Clusters of Orthologous Groups (COG/KOG) functional classifications.

Carbohydrate-active enzymes (CAZymes) were annotated using run_dbcan (v2.0.11) [45] from the dbCAN2 toolkit through three complementary approaches: HMMER (v3.4) [46] searches against dbCAN Hidden Markov Model (HMM) databases, DIAMOND (v2.1.8) [47] searches against CAZyDB, and Hotpep peptide-based searches [48]. To ensure annotation reliability and minimize false positives, only genes annotated by at least two of the three methods were retained for the final CAZymes catalog. These were subsequently classified into major categories including Glycoside Hydrolases (GHs), Glycosyl Transferases (GTs), Carbohydrate Esterases (CEs), Auxiliary Activities (AAs), Polysaccharide Lyases (PLs), and Carbohydrate-binding Modules (CBMs).

### 2.9. Genome Visualization

Whole-genome visualizations were generated using Circos (v0.69-8) [49] to provide comprehensive views of chromosomal architecture and genomic features in concentric ring format. Tracks were arranged from outermost to innermost as follows: chromosome ideograms with size information, GC content distribution, gene density, repeat density, and intragenomic synteny relationships. To balance visualization resolution and graphical clarity, window-based statistics for GC content, gene density, and repeat sequence coverage were computed using BEDtools (v2.30.0) [50] and seqtk (v1.4, Li, H., https://github.com/lh3/seqtk, accessed on 10 March 2026) with appropriately sized sliding windows to ensure optimal spatial resolution and display effectiveness.

### 2.10. Comparative Genomics Analysis

To resolve the phylogenetic placement of *T. sanguinea* within Tremellales, eight representative fungal genomes were selected alongside *T. sanguinea* for comparative analyses based on orthologous genes. The taxon sampling included *T. fuciformis* and *T. mesenterica* representing the genus *Tremella*, *Naematelia sinensis* and *N. encephala* representing the genus *Naematelia*, *Phaeotremella fagi*, *P. pseudofoliacea* and *P. skinneri* representing the genus *Phaeotremella*, and *Cryptococcus neoformans* as the outgroup (Table S2). This sampling strategy encompasses most of the major lineages currently recognized within Tremellales, thereby enabling robust inference of the phylogenetic position of *T. sanguinea* based on taxonomically representative taxa across the order.

Genomic data for *T. mesenterica*, *N. encephala*, and the outgroup species *C. neoformans* [51] were downloaded from the Ensembl database, including CDS sequences, protein sequences, and GFF annotation files. Genomic data for *T. fuciformis* and *N. sinensis* were obtained from NCBI as raw sequences requiring re-annotation. Genome assemblies for the three *Phaeotremella* species (*P. fagi*; *P. pseudofoliacea*; *P. skinneri*) and for *N. sinensis* lacked public gene annotations and were annotated in this study with the same pipeline. The re-annotation workflow included: (1) RNA-seq data quality control and alignment using HISAT2 (v2.2.1) [39] to map transcriptomic reads to genomes; (2) initial gene prediction using BRAKER (v2.1.6) [40]; (3) transcript assembly using rnaSPAdes (SPAdes v3.15.5) [42], followed by final gene annotation using MAKER (v3.1.3) [41] integrated with homologous protein sequences; and (4) functional annotation using eggNOG-mapper (v2.1.12) [44] to obtain GO, KEGG, and COG/KOG classifications.

#### 2.10.1. Gene Family Identification and Phylogenetic Analysis

Orthologous gene families among the nine species were identified using OrthoFinder (v2.5.4) [52] based on DIAMOND (v2.1.8) [47] rapid sequence searching and MAFFT (v7.525) [53] multiple sequence alignment. Species phylogenetic relationships were validated through both concatenation and coalescent approaches: maximum likelihood phylogenetic trees were constructed using raxmlHPC-PTHREADS (v8.2.13) [54], and species tree inference was performed using ASTRAL (v5.7.8) [55] based on gene trees.

#### 2.10.2. Divergence Time Estimation and Gene Family Evolution

Divergence times were estimated using the MCMCTree program in the PAML (v4.9) [56] package, calibrated with fossil constraints from the TimeTree database [57]. A single calibration was applied at the root node (soft bounds 140–170 Ma). Divergence times were estimated under a strict clock; relaxed-clock models were not identifiable under this single-calibration scheme and were therefore not used. Gene family size dynamics were simulated using CAFE (v4.2.1) [58] to identify expansion and contraction events across different lineages.

#### 2.10.3. CAZyme Annotation and Comparison

CAZyme annotation of the nine fungal genomes was performed using dbCAN2 [45] (v4.0) with an identical parameter set for all nine genomes (--tools hmmer diamond), retaining assignments supported by at least two tools. All annotated CAZymes were subsequently classified into six major functional classes—glycoside hydrolases (GH), glycosyltransferases (GT), polysaccharide lyases (PL), carbohydrate esterases (CE), auxiliary activities (AA), and carbohydrate-binding modules (CBM)—and their proportional compositions were visualized as stacked bar charts. This overview enabled a macroscopic comparison of the capacity for polysaccharide degradation, modification, and biosynthesis across the nine species (Table S3).

At the subfamily level, CAZyme subfamilies with a maximum gene copy number of ≥3 in at least one of the nine species were retained, yielding 41 subfamilies for downstream analysis. Gene copy numbers were subjected to row-wise Z-score normalization to eliminate the confounding effect of absolute abundance differences among subfamilies. Within each class, subfamilies were ordered by the difference between the *Phaeotremella* mean and the mean of the remaining genomes. All 41 subfamilies across the nine genomes were visualized as a heatmap using SciPy (v1.10.1) and Matplotlib (v3.7.1) [59] in Python (v3.10) to reveal inter-species variation in CAZyme subfamily composition.

#### 2.10.4. GO Enrichment of Gene-Family Sets

Gene Ontology (GO) functional annotation was performed on the predicted protein sequences of all nine species. To test whether gene families that expanded or contracted on particular branches share functional themes, GO enrichment was carried out at the level of gene families rather than individual genes. Each orthogroup was assigned the union of the GO terms carried by its member genes across all nine species; the background comprised all orthogroups with at least one GO term. Enrichment of the CAFE-inferred expanded or contracted family sets was assessed with the hypergeometric test and Benjamini–Hochberg FDR control (significance at FDR < 0.05) [60]. GO terms that are not applicable to fungi and arise from ortholog-based annotation transfer (for example, transforming-growth-factor-β receptor signalling, epithelial–mesenchymal transition and digestive-system processes) were excluded prior to testing; the exclusion list is provided as Table S5.

#### 2.10.5. Synteny and Whole-Genome Duplication Analysis

Intragenomic synteny of the *T. sanguinea* assembly was analysed with JCVI (v1.3.6) [61] using the MCScan pipeline; the resulting syntenic blocks are shown as the innermost ribbon track of the Circos plot.

To test for a whole-genome duplication (WGD) event in this lineage, rates of synonymous substitution (Ks) were estimated for intraspecific paralogous gene pairs with wgd [v1.1.1], https://github.com/arzwa/wgd, accessed on 10 April 2026). Paralogous pairs were identified from an all-versus-all comparison of the predicted proteome and clustered into gene families; each pair was aligned at the protein level, the alignment was back-translated into the corresponding codon alignment, and Ks was estimated by maximum likelihood as implemented in the pipeline. Pairs with Ks outside the interval [0.01, 5] were discarded, since very low values reflect recent tandem duplication and very high values are subject to saturation at synonymous sites. The distribution of Ks across the retained pairs was then examined for a secondary peak, which is the signature of the synchronous burst of gene duplication produced by a WGD. Because Ks records the age of gene duplication rather than the time of species divergence, this analysis is interpreted only with respect to the presence or absence of a duplication event within the lineage, and not as evidence bearing on divergence times.

#### 2.10.6. Secondary Metabolite Gene Clusters and Pigment Biosynthesis Pathways

Biosynthetic gene clusters (BGCs) were predicted with antiSMASH (fungal version 8.0.4, web server) [62] using the relaxed detection strictness; cluster types and boundaries were taken directly from the antiSMASH output. This analysis was performed on six of the nine genomes—*T. sanguinea*, the three *Phaeotremella* species, *T. fuciformis* and *N. sinensis*—which together span the *Phaeotremella* clade and the two most closely related sampled lineages; *T. mesenterica*, *N. encephala* and *C. neoformans* were not included. Because BGC detection is sensitive to assembly contiguity, clusters lying at contig termini in the scaffold-level assemblies may be truncated or missed; cluster counts are therefore compared qualitatively only.

To assess the genetic basis of the distinctive basidiocarp pigmentation, reference proteins of three fungal pigment pathways were retrieved from UniProt: DHN-melanin (alb1/pksP, arp1, arp2 and ayg1 of *Aspergillus fumigatus*), DOPA-type melanin (laccases LAC1 and LAC2 of *Cryptococcus* spp., abr1 and abr2 of *A. fumigatus*, and polyphenol oxidases PPO1–PPO4 of *Agaricus bisporus*) and carotenoid biosynthesis (crtYB, crtI, crtE, crtS and crtR of *Phaffia rhodozyma*). Each reference protein was searched against the predicted proteomes with BLASTP (v2.15.0) [63] under a stringent threshold (E ≤ 1 × 10^−20^, ≥40% identity, ≥60% query coverage) and a relaxed threshold (E ≤ 1 × 10^−5^). *C. neoformans* H99, in which both melanin pathways are characterised, was included as a positive reference, giving seven proteomes for this analysis. Hits to genes belonging to large, promiscuous superfamilies (arp2 and THN reductase, short-chain dehydrogenase/reductase superfamily; crtS and crtR, cytochrome P450 and P450 reductase families) were flagged as non-specific and excluded from interpretation (Table S8).

## 3. Results

### 3.1. Sequencing Data Generation and Coverage Statistics

To build a high-depth multi-omics dataset for *T. sanguinea*, we employed a combination of short-read sequencing, long-read sequencing, and chromatin conformation capture technologies. The sequencing depths achieved across all platforms substantially exceeded initial estimates, providing ample coverage for telomere-to-telomere (T2T) genome assembly.

Short-read whole-genome sequencing (WGS) was performed on the DNBSEQ-T7 platform (MGI, Shenzhen, China), generating 12.44 Gb of raw data and 12.4 Gb of high-quality clean data (~578×) from a total of 41,462,825 reads. Long-read sequencing was carried out on the ONT PromethION platform, yielding 8.93 Gb of raw data and 8.66 Gb of effective data (213,952 effective reads, ~404×). Hi-C chromatin conformation capture sequencing produced 10.5 Gb of raw data and 10.4 Gb of clean data (~35 million high-quality read pairs, ~485×). In addition, RNA sequencing was performed on the MGI DNBSEQ platform, generating 34.2 Gb of raw data (~230.65 million reads) to support accurate gene model prediction (Table 1). This high-coverage, multi-platform sequencing strategy enabled effective resolution of complex repetitive regions and provided the foundation for constructing a gap-free physical map of the genome.

### 3.2. T2T Assembly Quality, Structural Validation, and Telomere Integrity

Deep integration of multi-source sequencing data enabled the generation of a seamless, full-length T2T assembly for *T. sanguinea*. The assembled genome spans 21.44 Mb across 9 gap-free chromosomes, with the largest chromosome (Chr01) measuring 5.84 Mb and the smallest (Chr09) measuring 0.27 Mb. The assembly achieved an N50 of 5,045,101 bp (~5.05 Mb), an N90 of 1,436,535 bp, an L50 of 2, zero gaps, and a GC content of 51.49% (Table 2). The quality value (QV) of 58.51 indicates high base-level accuracy (Table 2). BUSCO assessment against the fungi_odb10 database revealed 98.0% genome completeness (C: 98.0% [S: 97.9%, D: 0.1%], F: 0.3%, M: 1.7%), confirming near-complete representation of conserved fungal gene content (Table 2).

Chromosome termini were verified by telomere motif analysis, which consistently identified AACCCCCT/AGGGGGTT as the telomeric repeat unit, consistent with the canonical telomere sequences reported in Basidiomycota. Following telomeric extension using FungalTeloExtender, all 18 termini across the 9 chromosomes were successfully extended, achieving 100% telomere coverage (18/18) (Table 2, Table 3 and Table S1). Extension lengths ranged from 98 bp (Chr08, 5′ end) to 665 bp (Chr01, 3′ end) (Table S1).

**Figure 2:**
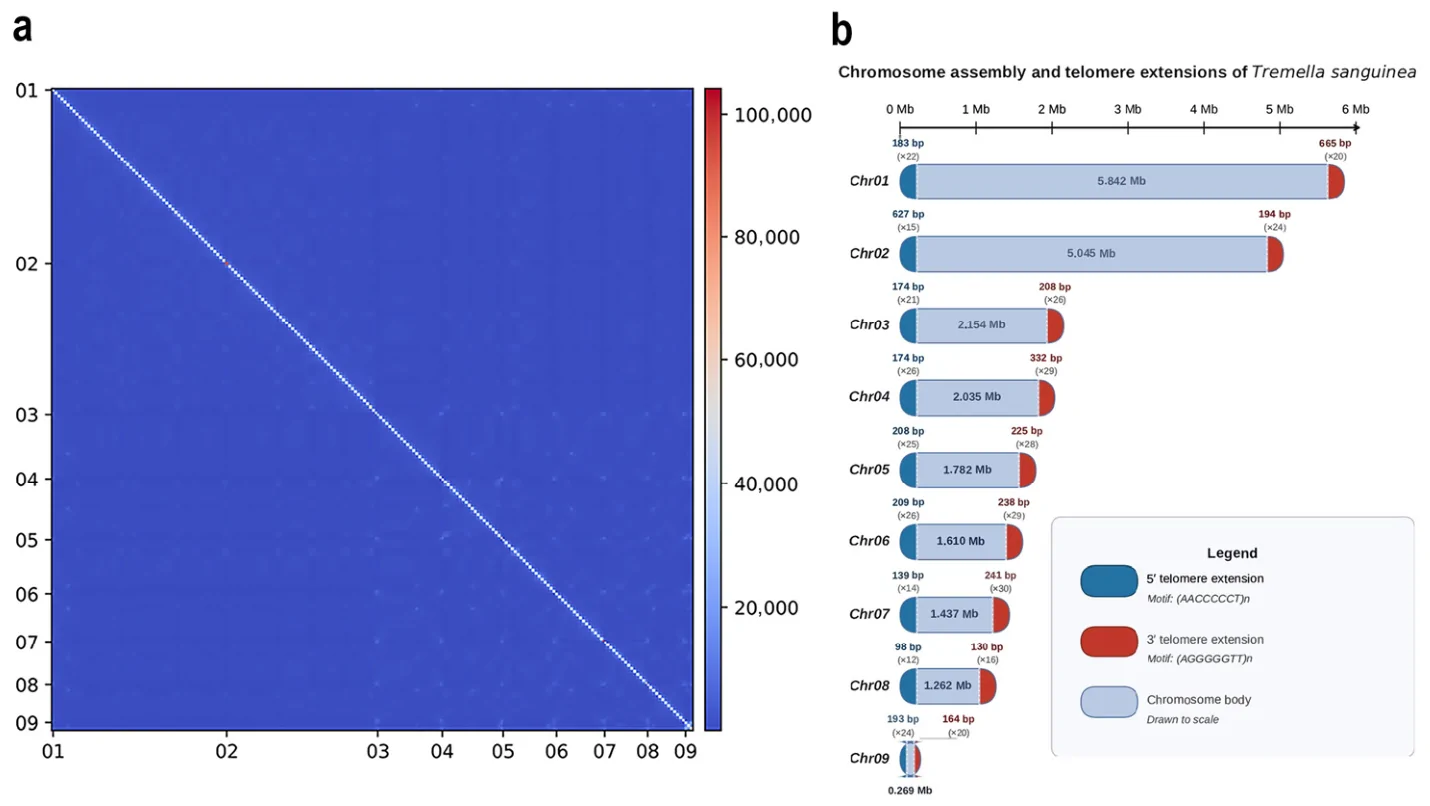
Chromosome-level genome assembly and telomere characterization of *T. sanguinea*. (**a**) Whole-genome Hi-C contact heatmap at 100 kb resolution confirming chromosome-scale structural integrity of the nine chromosomes. (**b**) Chromosome assembly and telomere extension diagram of *T. sanguinea* drawn to scale, showing 5′ telomere extensions (AACCCCCT)*n* and 3′ telomere extensions (AGGGGGTT)*n* for each chromosome, with telomere lengths and repeat counts indicated.

The Hi-C contact heatmap (Figure 2a and Figure S1) revealed strong cis-chromosomal interaction signals distributed along the diagonal at the whole-genome scale, with negligible trans-chromosomal (inter-chromosomal) contact signals, clearly delineating the nine topologically independent chromosomal domains of *T. sanguinea* and providing robust validation of the chromosome-scale assembly.

Telomere extension visualization (Figure 2b) illustrated the complete telomeric capping of all nine chromosomes at both termini: 5′ ends are highlighted in teal (motif: AACCCCCT) and 3′ ends in red (motif: AGGGGGTT), with extension lengths annotated for each chromosome. All 18 chromosomal termini were successfully capped, confirming 100% telomere integrity across the genome.

The genome survey provides a direct test of the ploidy and genotypic composition of the sequenced material. The 21-mer spectrum computed from 12.4 Gb of quality-filtered short reads contains a single coverage peak, at 454×, and no peak at half that coverage (Figure S2). This is a property of the read data rather than of any fitted model, and it is the expected signature of a single haploid genotype: a dikaryon, or a pool of meiotically distinct sibling basidiospores, would necessarily generate a second peak at half the main coverage, because allelic k-mers would be present at half the depth of shared ones. Models fitted to this spectrum returned haploid genome lengths of 18.90–18.93 Mb and 20.92–20.96 Mb, bracketing the 21.44 Mb of the final assembly. Both estimates fall at or below the assembled size, as expected of k-mer-based estimation, because high-copy tandem arrays such as the rDNA cluster and the telomeric and subtelomeric repeats are collapsed in k-mer space but fully resolved in a T2T assembly; a second, divergent haplotype would inflate rather than deflate these estimates.

Three features of the assembly itself are consistent with the same conclusion. First, only 0.1% of BUSCO genes were duplicated (C: 98.0% [S: 97.9%, D: 0.1%]), whereas dikaryotic or genotypically mixed input characteristically inflates this fraction, because divergent allelic copies are retained as separate contigs. Second, the data resolve into exactly nine gap-free chromosomes with telomeric repeats at all 18 termini and a consensus quality value of 58.51, whereas allelic divergence within a sample typically fragments the assembly graph, produces haplotype switching and inflates total assembly length. Third, the Hi-C contact map shows nine mutually exclusive interaction blocks with no secondary off-diagonal signal of the kind produced by unresolved allelic duplicates (Figure 2a and Figure S1). The assembly reported here is therefore the physical map of a monokaryotic, haploid genome, directly comparable with the haploid reference genomes of *T. fuciformis* and *N. sinensis* used for comparison in this study.

**Figure 3:**
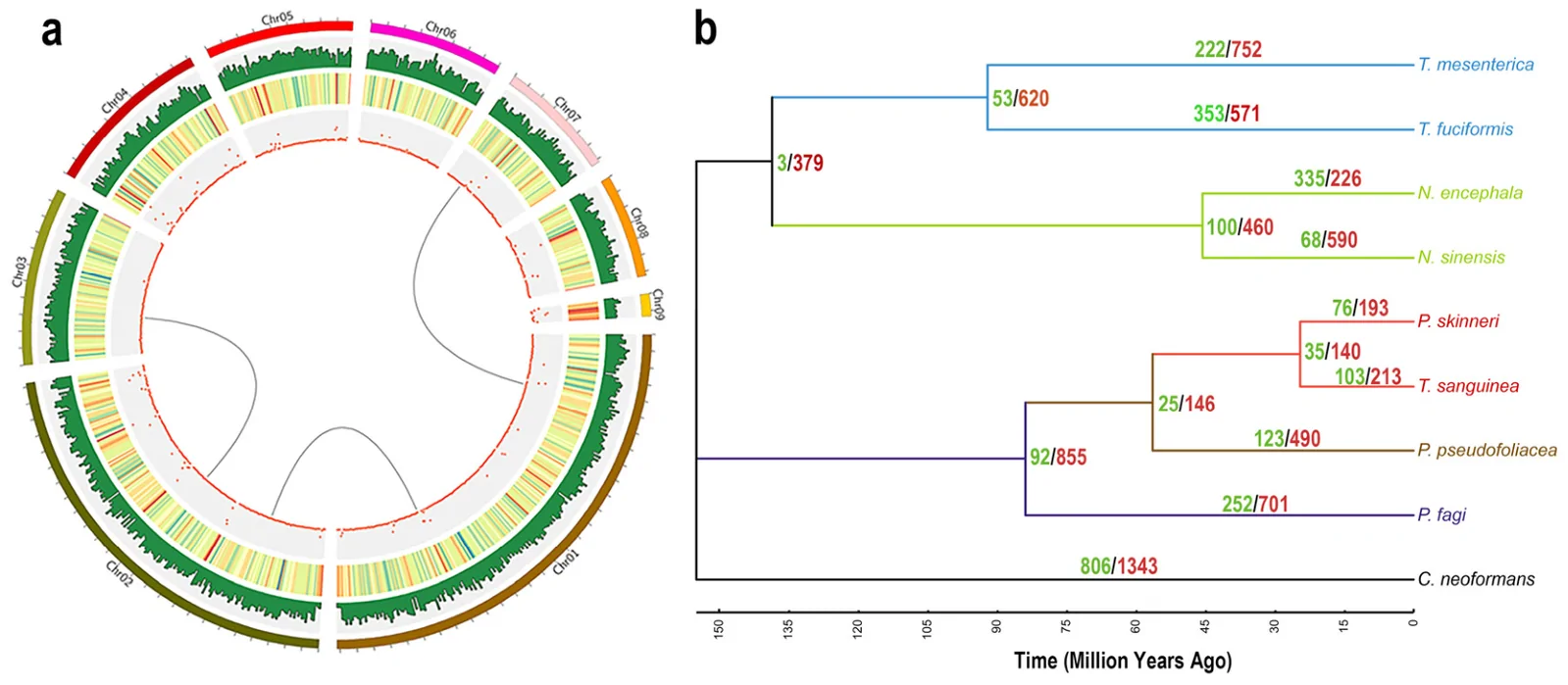
Genome-wide chromosomal characteristics and phylogenetic analysis of *T. sanguinea*. (**a**) Circos plot displaying genomic features from outer to inner rings: chromosome ideograms (Chr01–Chr09), GC content distribution, gene density, repeat density, and intra- and inter-chromosomal synteny ribbons. (**b**) Time-calibrated phylogeny of *T. sanguinea* and related species inferred from single-copy orthologs using maximum likelihood and MCMCTree, with *C. neoformans* as outgroup. Numbers at each node indicate gene family expansions (green) and contractions (red) estimated by CAFE analysis. Expansions and contractions are inferred relative to the reconstructed ancestral state at the parent node, not relative to other extant species. Time scale is in million years ago (Mya).

The multi-layered Circos plot (Figure 3a) displays, from the outermost to the innermost tracks, GC content distribution, gene density, repeat element distribution, and intragenomic synteny relationships across the nine chromosomes of *T. sanguinea*. All tracks exhibited broadly uniform distributions without apparent regional enrichment or depletion, indicative of a highly stable genomic architecture. Notably, Chr09, as the smallest chromosome, showed subtle differences in gene density and repeat element proportion relative to the remaining chromosomes, which may be attributable to its considerably smaller physical size. Taken together, these results demonstrate that the *T. sanguinea* T2T genome assembly satisfies stringent quality criteria at both the levels of structural integrity and sequence accuracy, establishing it as a robust reference for downstream analyses.

### 3.3. Repetitive Element Landscapes and Functional Annotation

The repetitive element content of the *T. sanguinea* genome was relatively conserved. Interspersed repeats totaled 593,981 bp in length, accounting for only 2.77% of the genome, with retroelements representing the predominant component (548 elements, 461,247 bp, 2.15% of the genome). Unclassified repetitive elements comprised 661 elements spanning 132,734 bp (0.62%), while simple repeats and low-complexity regions accounted for 3468 elements (164,412 bp, 0.77%) and 958 elements (50,914 bp, 0.24%) of the genome, respectively (Table 4). Overall, the repeat element composition of *T. sanguinea* is relatively simple, with limited transposable element expansion, reflecting a compact genomic architecture.

A hybrid annotation pipeline was employed to generate a high-quality protein-coding gene set for *T. sanguinea*. A total of 6949 genes were predicted, including 6358 protein-coding genes, with an average gene length of 2675.24 bp, an average of 7.59 exons per gene, and an average intron length of 77.47 bp. The total numbers of exons and introns were 52,733 and 45,784, respectively (Table 5). Functional annotation revealed that 87.78% of genes were successfully assigned to COG orthologous clusters, 56.67% received KEGG KO functional annotations, 36.08% were mapped to KEGG metabolic pathways, and 50.85% were associated with GO terms (Table 5). Collectively, these results demonstrate that the constructed gene set is both highly complete and functionally comprehensive, providing a robust foundation for subsequent comparative and functional genomic analyses.

### 3.4. Orthologous Group Identification and Genome Comparability

To elucidate the phylogenetic position of *T. sanguinea* within the order Tremellales, comparative genomic analyses were conducted across nine representative fungal genomes: *T. fuciformis* and *T. mesenterica* (*Tremella*), *N. sinensis* and *N. encephala* (*Naematelia*), *P. fagi*, *P. pseudofoliacea* and *P. skinneri* (*Phaeotremella*), *T. sanguinea*, and *C. neoformans* as outgroup (Table S2). This sampling scheme spans the major known clades of Tremellales, ensuring that the phylogenetic placement inferred for *T. sanguinea* is evaluated relative to a taxonomically representative cross-section of the order. All nine proteomes showed comparable BUSCO completeness (92.1–96.7%, fungi_odb12, protein mode), and orthogroup coverage fell within a narrow range (77.9–86.2% of the 7245 orthogroups), so gene-content comparisons are not confounded by differences in annotation completeness. Protein sequences from all nine species were clustered into orthologous gene families using OrthoFinder. Statistics for total proteins, genes assigned to orthogroups, unassigned genes, detected orthogroups, and species-specific orthogroups/genes for each taxon are summarised in Table 6.

At the species level, *T. mesenterica* carried the largest number of gene models (8291) and by far the largest fraction of unassigned genes (1236; 14.9%), consistent with its fragmented 7.44× draft assembly; *C. neoformans*, as the outgroup, showed the largest number of species-specific orthogroups (125). By comparison, *T. sanguinea* exhibited zero species-specific orthogroups and species-specific genes (Table 6), revealing strong genomic conservation relative to the other sampled taxa. Across the nine genomes, 3312 orthogroups were single-copy in all species. *T. sanguinea* contained exactly one gene in 5655 orthogroups, comparable to *N. encephala* (5680), *P. skinneri* (5669) and *N. sinensis* (5627). The abundant single-copy orthologous genes support high accuracy of coding sequences within the T2T genome assembly and supply robust molecular markers for downstream phylogenetic reconstruction and divergence time estimation.

Gene family evolution analysis (Figure 3b; Table 7) showed that *T. sanguinea* experienced 103 expansions (128 genes gained) and 213 contractions (221 genes lost), resulting in a net change rate of −0.0128. A total of 4 gene families exhibited rapid expansion, while 4 gene families showed rapid contraction. By comparison, *T. fuciformis* showed a slightly positive mean rate of change (+0.013), driven by larger per-family gene gains (691 genes gained across 353 expansions vs. 597 genes lost across 571 contractions), whereas *T. mesenterica* experienced substantial gene family turnover (222 expansions vs. 752 contractions).The outgroup *C. neoformans* underwent expansion in 806 gene families (gaining 1153 genes) and contraction in 1343 gene families (losing 1447 genes), further substantiating its substantial genomic divergence from Tremellales species. At ancestral internal nodes, the common ancestor of *Tremella* and *Naematelia* (node <13>) experienced only 3 expansions and 379 contractions, whereas the *Tremella* crown node (node <11>) showed 53 expansions and 620 contractions. The *Phaeotremella* crown node (node <3>), representing the common ancestor of *P. fagi*, *P. pseudofoliacea*, *P. skinneri*, and *T. sanguinea*, underwent 92 expansions and 855 contractions—the strongest contraction signal observed across the entire tree—suggesting that extensive gene family remodeling occurred during the early diversification of the *Phaeotremella* lineage (Table 7).

### 3.5. Phylogenetic Reconstruction, Divergence Time Estimation, and Taxonomic Placement of T. sanguinea

Maximum-likelihood (RAxML) and coalescent (ASTRAL) analyses of single-copy orthologues recovered congruent topologies. In both, *T. sanguinea* was nested within *Phaeotremella* rather than within *Tremella* sensu stricto, forming a strongly supported sister relationship with *P. skinneri*; this clade was successively sister to *P. pseudofoliacea* and then to *P. fagi* (Figure 3b). ITS-based phylogenetic analysis (ITS4/ITS5; GenBank accession PV1770701) placed *T. sanguinea* within the same *Phaeotremella* clade, consistent with the genome-scale tree. However, the ITS topology did not confidently resolve the sister relationship between *T. sanguinea* and *P. skinneri*, underscoring the value of genome-wide data for robust species-level phylogenetic inference.

Divergence-time estimation placed the split between *C. neoformans* and the Tremellales at 154.90 MYA (95% HPD 139.98–169.99). Within Tremellales, the *Tremella* + *Naematelia* lineage diverged at 138.58 MYA (125.23–152.06), and the *Phaeotremella* crown group at 83.84 MYA (75.77–92.02). Within *Phaeotremella*, *P. fagi* diverged first, followed by *P. pseudofoliacea* at 56.39 MYA (50.96–61.89), with *T. sanguinea* and *P. skinneri* diverging most recently, at 24.50 MYA (22.14–26.89). For comparison, *T. fuciformis* and *T. mesenterica* diverged at 92.04 MYA (83.17–101.00), and *N. encephala* and *N. sinensis* at 45.60 MYA (41.21–50.05) (Figure 3b).

### 3.6. Carbohydrate-Active Enzyme (CAZyme) Repertoire

#### 3.6.1. Overall CAZyme Composition

A total of 185 CAZyme-encoding genes were identified in *T. sanguinea*: GH 43.2%, GT 39.5%, AA 7.0%, CBM 4.3%, CE 3.2% and PL 2.7%. GH and GT together accounted for 80–85% of the annotated CAZymes in all nine genomes.

**Figure 4:**
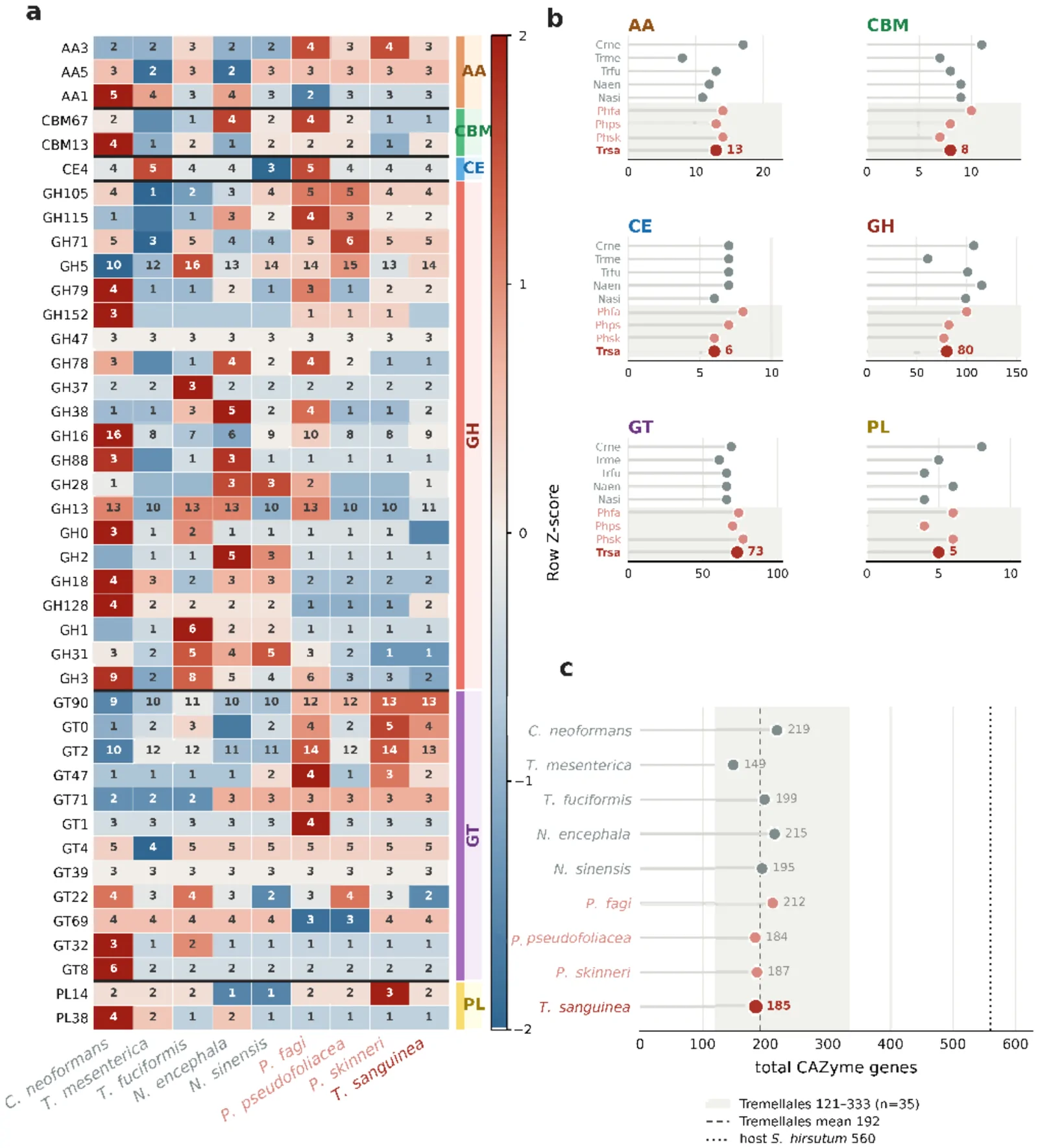
Comparative analysis of CAZyme repertoires across nine Tremellales genomes. Species are ordered by phylogeny (*C. neoformans*|*T. mesenterica*, *T. fuciformis*, *N. encephala*, *N. sinensis*|*P. fagi*, *P. pseudofoliacea*, *P. skinneri*, *T. sanguinea*); *T. sanguinea* is highlighted throughout. (**a**) Heatmap of 41 CAZyme subfamilies retained after filtering (maximum copy number ≥ 3 in at least one genome). Colours show row-wise Z-scores clipped at ±2; numbers within cells are raw gene copy numbers. Subfamilies are grouped by CAZyme class (AA, CBM, CE, GH, GT, PL; colour bars at right) and, within each class, ordered by the difference between the *Phaeotremella* mean and the mean of the remaining genomes. blank cells indicate zero copies. (**b**) Gene copy number of the six major CAZyme classes; the shaded band marks the four *Phaeotremella* genomes and values are labelled for *T. sanguinea* and for the per-class maximum. (**c**) Total CAZyme content of each genome, shown against the range reported for Tremellales (121–333 genes; *n* = 35 strains; grey band, with the mean of 192 as a dashed line) and against the content of the fungal host *Stereum hirsutum* (560 genes; dotted line).

Total CAZyme content ranged from 149 (*T. mesenterica*) to 219 (*C. neoformans*), with *T. sanguinea* at 185, i.e., seventh of nine and well within the range reported for Tremellales [64] (121–333; *n* = 35). The CAZyme repertoire of *T. sanguinea* is therefore unremarkable within the order (Figure 4b,c).

#### 3.6.2. Subfamily-Level Distribution

At the subfamily level, 41 subfamilies with a maximum gene copy number of ≥3 across the nine species were retained (Figure 4a). Species were ordered by phylogeny rather than alphabetically, so that clade-level patterns are directly visible.

To place the CAZyme content of the nine genomes in a broader context, two published values are shown for reference in Figure 4c: the range of CAZyme counts reported across 35 Tremellales strains (121–333, mean 192) [65], and the CAZyme content of Stereum hirsutum, the fungal host of both *T. sanguinea* and *N. sinensis* (560) [18]. These values were obtained with different dbCAN versions and detection thresholds from those used here, and are therefore used only for order-of-magnitude comparison, not as a common scale.

Two subfamilies showed complete separation between *Phaeotremella* and the remaining five genomes: GT90 was consistently higher in *Phaeotremella* (12–13 copies vs. 9–11 elsewhere), whereas GH20 (β-hexosaminidase) was absent from all four *Phaeotremella* genomes but present as a single copy in each of the other five. GH20 is not shown in Figure 4a because it did not meet the filtering threshold (max copy number = 1, threshold ≥ 3). No other subfamily separated the two groups without overlap.

Normalised per 1000 predicted proteins, the four *Phaeotremella* genomes occupied the four highest positions for glycosyltransferase density (*P. skinneri* 11.6, *P. fagi* 11.2, *P. pseudofoliacea* 10.8 and *T. sanguinea* 10.5, against 7.4–10.2 in the remaining five genomes), with GT2 and GT90 the principal contributors. This is consistent with the abundant extracellular polysaccharide that forms the gelatinous basidiocarp of these fungi and to which the reported bioactivities of *T. sanguinea* are attributed [8,9,10], although the association is one of gene content with phenotype and does not by itself demonstrate a functional link.

#### 3.6.3. Secondary Metabolite Gene Clusters and the Genetic Basis of Pigmentation

AntiSMASH predicted four to eight BGC regions per genome across the six genomes examined (Tables S6 and S7). Terpene and terpene-precursor clusters were the dominant class in every genome, and a clavaric acid-like terpene cluster was detected with high similarity confidence in all six, indicating a terpenoid capacity that is conserved across Tremellales. *T. sanguinea* carried seven BGC regions—three terpene, two terpene-precursor, one T3PKS and one NRPS-like—a complement indistinguishable in number and composition from that of *P. skinneri* and *P. fagi*.

One cluster type showed a clade-restricted distribution. A type III polyketide synthase (T3PKS) cluster was present as a single copy in all four members of the *Phaeotremella* clade, with a closely comparable span in each (61.4–61.7 kb), and was absent from both non-*Phaeotremella* genomes examined. The direction of this inference is robust to assembly quality: the cluster is observed as present in three fragmented scaffold assemblies, where presence is unaffected by contiguity, and as absent in two chromosome-level assemblies, where absence is meaningful. Two limitations should nonetheless be stated. First, because the four *Phaeotremella* genomes share the cluster by common descent, this distribution records a single evolutionary event rather than four independent observations, and only two genomes outside the clade were examined. Second, the three congeners carrying the cluster are uniformly brown, whereas *T. sanguinea* is dark reddish-brown and exudes an amber-red pigment, yet all four carry exactly one copy; the cluster therefore cannot by itself account for what distinguishes *T. sanguinea*. We report it as a candidate clade-diagnostic locus awaiting chemical characterisation, not as a determinant of any phenotype.

Two independent lines of evidence indicate that the dark pigmentation of *T. sanguinea* is not produced by the DHN-melanin pathway. No type I (non-reducing) PKS was detected by antiSMASH in any of the six genomes examined, and the pathway-specific genes alb1/pksP, arp1 and ayg1 returned no hit in any of the seven proteomes searched, including that of *C. neoformans*, in which melanin biosynthesis is characterised (Table S8). The 16–18 hits recovered by arp2 and THN reductase in every genome represent the short-chain dehydrogenase/reductase superfamily background and are not evidence for the pathway. Homologues of the multicopper oxidase/laccase family associated with DOPA-type melanin were recovered from every genome, but *T. sanguinea* carried no more of them (three) than its congeners (two to three) and fewer than *C. neoformans* (five), and no Agaricus bisporus-type polyphenol oxidase (PPO1–PPO4) was detected in any genome. The presence of this family therefore does not by itself account for the pigmentation that distinguishes *T. sanguinea* from the uniformly brown members of its clade.

Carotenoid biosynthesis could not be resolved by homology search in this dataset. crtYB, which encodes the committed bifunctional phytoene synthase/lycopene cyclase, returned no hit passing the stringent threshold in any genome—including that of the orange-yellow *N. sinensis*, in which carotenoid pigmentation is expected. The positive control therefore failed, and the negative result obtained for *T. sanguinea* carries no information with respect to this pathway. Identification of the amber-red pigment of *T. sanguinea* will require characterisation at the metabolite level, which lies beyond the scope of the present study.

### 3.7. Gene Family Evolution and Functional Enrichment

#### 3.7.1. Gene Family Expansion and Contraction Across the Nine Genomes

Gene family evolution was reconstructed with CAFE across the nine genomes (Table 7). Contractions outnumbered expansions on most branches. The strongest signal was at the *Phaeotremella* crown node, which showed 92 expansions against 855 contractions (mean change −0.1042, the most negative in the tree). On the *T. sanguinea* branch, 103 families expanded and 213 contracted.

No coherent functional enrichment was recovered for the contracted family sets. Of the 855 families contracted at the *Phaeotremella* crown node, only 101 (11.8%) carried any GO annotation, compared with 48.8% of orthogroups overall, indicating that the families lost on this branch are disproportionately lineage-specific and functionally uncharacterised.

#### 3.7.2. Functional Enrichment of the Families Expanded in *T. sanguinea*

**Figure 5:**
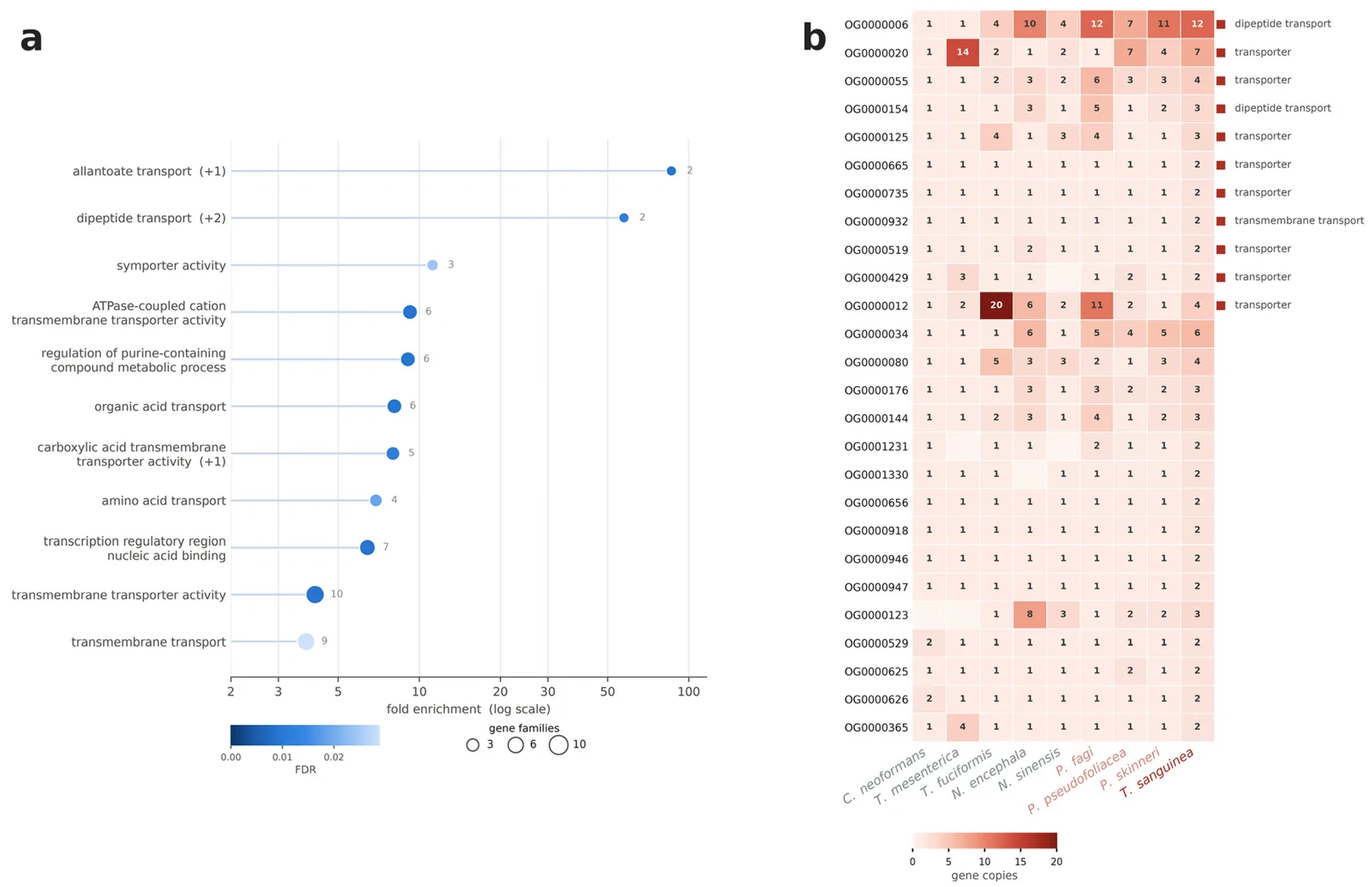
Functional characterisation of the gene families that expanded on the *T. sanguinea* branch. (**a**) GO enrichment of the CAFE-inferred expanded families. The unit of testing is the gene family, not the gene: each orthogroup was assigned the union of the GO terms carried by its member genes across all nine genomes, and the background comprised all orthogroups with at least one GO term (3534 of 7245). Enrichment was assessed with the hypergeometric test and Benjamini–Hochberg FDR control; all terms shown are significant at FDR < 0.05. The *x*-axis gives fold enrichment on a logarithmic scale, dot size the number of families contributing to the term, and dot colour the FDR. GO terms that are not applicable to fungi and arise from ortholog-based annotation transfer were excluded before testing (Table S4). Terms supported by an identical set of families were collapsed to a single representative, with the number of merged terms indicated as “(+N)”; the full mapping is given in Table S5. (**b**) Gene copy number, across the nine genomes, of the 26 expanded families that carry at least one significantly enriched GO term (of 103 families expanded on this branch in total). Rows are ordered with transport-related families first; red squares mark the 11 families carrying transport-related GO terms, annotated with the corresponding term. Species are arranged in phylogenetic order and cell values are raw gene copy numbers. Expansions and contractions are inferred relative to the reconstructed ancestral state at the parent node, not relative to other extant species.

Functional enrichment of the CAFE-inferred family sets was tested at the level of gene families. Of the 103 families expanded on this branch, 41 carried at least one GO term and constituted the test set; 26 of these carried a term that remained significant after FDR correction, and 11 of the 26 were transport-related. Families expanded on the *T. sanguinea* branch were significantly enriched for transport-related functions (FDR < 0.05): transmembrane transporter activity (10 families), transcription regulatory region nucleic acid binding (7), ATPase-coupled cation transmembrane transporter activity (6), organic acid transport (6), carboxylic acid transmembrane transporter activity (5), and, at lower family counts, dipeptide transport and allantoate transport (Figure 5a). Of the 103 expanded families, 26 carried a significantly enriched GO term and 11 of these were transport-related (Figure 5b).

### 3.8. Intragenomic Synteny and Absence of Recent Whole-Genome Duplication

To assess whether *T. sanguinea* underwent whole-genome duplication (WGD), synonymous substitution rates (Ks) of intraspecific paralogous gene pairs were calculated using wgd. A total of 188 paralogous pairs were identified, with Ks values showing a broad unimodal distribution peaking at Ks > 2, with no sharp peak indicative of WGD. The Ks values of the retained paralogous pairs are concentrated above 2, indicating that these duplications are ancient and that synonymous sites are approaching saturation, which limits the resolution of Ks-based detection for old events. Together with the compact genome of *T. sanguinea* (21.44 Mb), these results indicate the absence of a recent whole-genome duplication in this lineage.

## 4. Discussion

In this study, we present the first T2T-level genome assembly of *T. sanguinea*, yielding 9 gap-free chromosomes with a total genome size of 21.44 Mb, an N50 of 5.05 Mb, a QV of 58.51, and a BUSCO completeness score of 98.0%. Telomeric sequences (AACCCCCT/AGGGGGTT) were successfully identified and extended at all 18 chromosome ends, achieving 100% telomere coverage. This high-quality genomic resource fills a notable gap in available genome data for Tremellales, enabling, for the first time, precise phylogenetic placement of *T. sanguinea* and cross-species comparative genomic analyses.

Phylogenetic reconstruction from single-copy orthologues placed *T. sanguinea* within *Phaeotremella*, as sister to *P. skinneri*, rather than within *Tremella* sensu stricto. Maximum-likelihood and coalescent analyses recovered the same topology, and the placement was recovered against a taxon sample that includes the type species of the genus, *P. pseudofoliacea*.

Divergence-time estimation dated the *T. sanguinea*–*P. skinneri* split to 24.50 MYA (95% HPD 22.14–26.89) and the *Phaeotremella* crown group to 83.84 MYA (75.77–92.02), both well nested within the Tremellales radiation rather than at its base.

Earlier multi-locus analyses had already indicated that this species belongs in *Phaeotremella* rather than in *Tremella* sensu stricto [7]. The present phylogenomic analysis, based on 3312 single-copy orthologues and including the type species of the genus, corroborates that placement at genome scale and provides the first divergence-time estimates for the lineage. This is consistent with the seven-gene phylogeny of Liu et al. [2], which showed *Tremella* sensu lato to be polyphyletic and led to the resurrection of *Phaeotremella*. Formally transferring the species requires a nomenclatural act that lies outside the scope of this genomic study; we therefore retain the name *T. sanguinea* throughout and refrain from proposing a new combination here.

Gene family evolution across the nine genomes was contraction-biased on most branches. On the *T. sanguinea* branch, 103 families expanded and 213 contracted (mean change −0.0128); the strongest signal in the tree was at the *Phaeotremella* crown node (92 expansions vs. 855 contractions, mean change −0.1042), indicating that gene loss on this branch preceded the divergence of *T. sanguinea* itself.

The CAZyme repertoire of *T. sanguinea* (185 genes) lies within the range reported for Tremellales [65] (121–333; *n* = 35) and is unremarkable relative to the other eight genomes (seventh of nine). By contrast, functional enrichment of the CAFE-inferred expanded families identified a coherent signal in transport: transmembrane transporter activity, cation and organic/carboxylic acid transport, and, more specifically, dipeptide and allantoate transport—the latter an intermediate of purine catabolism and hence a nitrogen source [64,66].

Together these observations suggest that nutrient acquisition in *T. sanguinea* relies on uptake of host-derived small molecules rather than on an expanded arsenal of hydrolytic enzymes. This is consistent with the haustorial mode of mycoparasitism characteristic of Tremellales, in which tremelloid haustorial cells penetrate the host cell wall and are thought to have an absorptive role [67], and with the genomic streamlining reported for *N. sinensis*, which parasitises the same host genus *Stereum* [18].

A comparable coupling of reduced degradative capacity with expanded nutrient-uptake capacity has been described in obligate biotrophic plant pathogens: rust fungi combine a contracted repertoire of plant-cell-wall-degrading enzymes with a markedly expanded set of oligopeptide and amino-acid transporters deployed at the haustorial interface [68,69,70]. Although the transporter families involved differ, the *T. sanguinea* genome suggests that an analogous trade-off may operate in fungus-parasitising Tremellales.

With respect to the properties for which *T. sanguinea* is valued, the genome identifies candidate loci rather than established determinants. The bioactivities reported for this species are attributed mainly to its polysaccharides [8,9,10], and the elevated glycosyltransferase density shared across the *Phaeotremella* clade provides a plausible genetic correlate of the abundant extracellular polysaccharide of the gelatinous basidiocarp. The seven biosynthetic gene clusters detected—predominantly terpene and terpene-precursor clusters, together with a T3PKS and an NRPS-like cluster—are consistent with the steroids, alkaloids and phenolic acids characterised chemically in this species [9], and a clavaric acid-like triterpene cluster conserved across Tremellales points to a terpenoid capacity retained throughout the order. None of these features is unique to *T. sanguinea*, and none has been linked experimentally to a specific metabolite or activity; they are reported here as targets for the chemical and expression work that such a link would require.

The genomic basis of the pigmentation for which this species is named remains unresolved. The absence of the DHN-melanin pathway, together with a complement of laccase-family genes no larger than that of its uniformly brown congeners, indicates that the amber-red pigment of *T. sanguinea* is unlikely to arise from either of the two melanin routes that account for dark pigmentation in most characterised fungi, while the homology-based approach used here could not resolve the carotenoid pathway even in a species whose basidiocarps are orange-yellow. Whether the clade-restricted T3PKS cluster contributes to the brown pigmentation shared across *Phaeotremella*, and what additional pathway underlies the red component unique to *T. sanguinea*, are questions that the present genome makes tractable but does not answer; both will require basidiocarp-stage transcriptomics or direct chemical characterisation of the pigment.

Convergent evolution of morphological characters in fungi frequently leads to misclassification; gelatinous basidiocarps in Basidiomycota have arisen independently on multiple occasions [71,72]. Molecular systematics has accumulated numerous cases of morphology-based classifications being revised within Tremellales: *N. sinensis* was transferred from *Tremella* to *Naematelia* [1,2]; *T. foliacea* was moved to the newly established genus *Phaeotremella* [2] with multiple cryptic species subsequently delineated [11]; and *Cryptococcus* was redefined based on multigene evidence [51]. In the present case, however, morphology and genome-scale phylogeny are in agreement, and it is the original generic assignment that was inconsistent with both. The generic name *Phaeotremella* refers to the dark-pigmented, foliose basidiocarps of its members; *T. sanguinea*, with dark reddish-brown to blackish-brown foliose basidiocarps, conforms to this morphology, whereas *Tremella* sensu stricto is typified by pale or brightly coloured species such as the white *T. fuciformis* and the yellow-orange *T. mesenterica*. The genomic placement recovered here therefore reconciles the macromorphology of *T. sanguinea* with its evolutionary position.

At the level of genomic resources, T2T gap-free assemblies effectively reduce gene loss and annotation bias caused by assembly fragmentation in conventional draft genomes, providing a more reliable data foundation for comparative genomics. As the cost of T2T sequencing continues to decline, telomere-complete chromosome-level assemblies are expected to become the standard in fungal phylogenomics. From an evolutionary perspective, the compact genome size (21.44 Mb) and moderate gene family dynamics of *T. sanguinea* are consistent with the streamlined genomes characteristic of Tremellales. Taxon sampling in this study covers nine genomes spanning *Tremella*, *Naematelia*, and *Phaeotremella*, including the type species of *Phaeotremella*. Nevertheless, only three of the approximately 30 currently recognised *Phaeotremella* species were included, and divergence time estimates relied solely on a single root calibration. Additional genome resources and improved calibration strategies incorporating fossil or secondary calibrations will further refine the evolutionary timescale and strengthen the phylogenetic resolution within Tremellales.

## 5. Conclusions

We report the first telomere-to-telomere genome for a member of *Phaeotremella*: nine gap-free chromosomes, 21.44 Mb, QV 58.51, with telomeric repeats at all 18 chromosome ends. Phylogenomic analysis of nine Tremellales genomes places *T. sanguinea* within *Phaeotremella*, as sister to *P. skinneri* (divergence 24.50 Ma), corroborating at genome scale the placement previously indicated by multi-locus data, and providing the first divergence-time estimates for the lineage. Gene family evolution was contraction-biased, most strongly at the *Phaeotremella* crown node, while families that expanded in *T. sanguinea* were enriched for transport functions—including dipeptide and allantoate transport—suggesting that this mycoparasite acquires host-derived nutrients through uptake rather than through an expanded hydrolytic repertoire.

## Figures and Tables

**Table 1:** Sequencing Data Statistics.

Species	Data Type	TotalBase of Raw Data (Gb)	TotalBase of Clean Data (Gb)	TotalReads of Raw Data	TotalReads of Clean Data	Coverage (×)
*T. sanguinea*	Short-read WGS	~12.44	~12.4	41,462,825	41,462,825	578×
*T. sanguinea*	Long-read	~8.93	~8.66	220,339	213,952	404×
*T. sanguinea*	Hi-C	~10.5	~10.4	35.1 M	35 M	485×
*T. sanguinea*	RNA-seq	~34.2	/	230.65 M	/	/

**Table 2:** Genome Assembly Statistics.

Feature	*T. sanguinea*
Assembly level	T2T
Total genome size (Mb)	21.44
Chromosome number	9
Largest chromosome (Mb)	5.84 (Chr01)
Smallest chromosome (Mb)	0.27 (Chr09)
Gap number	0
GC content (%)	51.49%
N50	5,045,101 bp
N90	1,436,535 bp
L50	2
L90	7
QV	58.5057
Telomere motif	AACCCCCT
Telomere-capped ends	18/18 (100%)
BUSCO (fungi_odb10)	98.00%
BUSCO breakdown	C: 98.0% [S: 97.9%, D: 0.1%], F: 0.3%, M: 1.7%

**Table 3:** Chromosome and Telomere Statistics.

Species	Chromosome	Length (bp)	Telomeres
*T. sanguinea*	Chr01	5,842,437	Both
*T. sanguinea*	Chr02	5,045,101	Both
*T. sanguinea*	Chr03	2,153,675	Both
*T. sanguinea*	Chr04	2,035,050	Both
*T. sanguinea*	Chr05	1,781,766	Both
*T. sanguinea*	Chr06	1,609,809	Both
*T. sanguinea*	Chr07	1,436,535	Both
*T. sanguinea*	Chr08	1,262,417	Both
*T. sanguinea*	Chr09	268,746	Both

**Table 4:** Repeat Element Statistics.

Element Type	Number of Elements	Length (bp)	Percentage of Sequence (%)
Total interspersed repeats	/	593,981	2.77%
Retroelements	548	461,247	2.15%
Simple repeats	3468	164,412	0.77%
Unclassified	661	132,734	0.62%
Low complexity	958	50,914	0.24%

**Table 5:** Gene Annotation Statistics.

Feature	*T. sanguinea*
Total Genes	6949
Protein-coding genes	6358
Average gene length (bp)	2675.24
Average exons per gene	7.59
Average intron length (bp)	77.47
Total exons	52,733
Total introns	45,784
Genes with GO annotation	50.85%
Genes with COG annotation	87.78%
Genes with KEGG KO annotation	56.67%
Genes with KEGG pathway annotation	36.08%

**Table 6:** Ortholog Statistics.

Species	Total Proteins	Genes in Orthogroups	Unassigned Genes	Orthogroups Present	Species-Specific Orthogroups	Species-Specific Genes
*C. neoformans*	7826	7369	457	6123	125	406
*T. mesenterica*	8291	7055	1236	5644	117	903
*T. fuciformis*	6979	6868	111	5749	7	20
*N. encephala*	7964	7144	820	6242	30	65
*N. sinensis*	6455	6399	56	5942	1	4
*P. fagi*	6592	6563	29	5767	1	2
*P. pseudofoliacea*	6468	6427	41	5881	0	0
*P. skinneri*	6611	6563	48	6035	0	0
*T. sanguinea*	6949	6588	361	6028	0	0

Note: Total gene counts should not be compared directly across annotation sources: the three publicly annotated genomes carry on average ~1350 more gene models than the six genomes annotated here with a uniform pipeline, despite showing slightly lower BUSCO completeness. Comparisons in this study are therefore based on orthogroup membership and on BUSCO-normalised metrics rather than on raw gene counts.

**Table 7:** Gene Family Evolution Summary.

Species/ Node	Expanded Families	Genes Gained	Unchanged Families	Contracted Families	Genes Lost	Average Rate of Change	Significant Expansions	Significant Contractions	Total Significant Changes
<11>	53	112	6572	620	626	−0.0709	17	1	18
Phps <8>	123	142	6632	490	502	−0.0497	5	9	14
Phfa <2>	252	372	6292	701	717	−0.0476	27	5	32
<3>	92	110	6298	855	865	−0.1042	3	5	8
Trsa <6>	103	128	6929	213	221	−0.0128	4	4	8
Trfu <12>	353	691	6321	571	597	0.013	62	10	72
Naen <14>	335	453	6684	226	235	0.0301	43	2	45
<13>	3	5	6863	379	380	−0.0518	1	0	1
<15>	100	118	6685	460	480	−0.05	5	8	13
Nasi <16>	68	79	6587	590	606	−0.0727	3	10	13
<7>	25	28	7074	146	149	−0.0167	3	2	5
<9>	0	0	7245	0	0	0	0	0	0
Trme <10>	222	1105	6271	752	824	0.0388	52	31	83
Crne <0>	806	1153	5096	1343	1447	−0.0406	21	9	30
<5>	35	40	7070	140	146	−0.0146	1	4	5
Phsk <4>	76	83	6976	193	201	−0.0163	3	6	9

Note: Rows are labelled with the species abbreviation followed by the CAFE node number; rows labelled with a number alone (<3>, <5>, <7>, <9>, <11>, <13>, <15>) are ancestral internal nodes. The CAFE labelled tree is (Crne <0>, ((Phfa <2>, ((Phsk <4>, Trsa <6>) <5>, Phps <8>) <7>) <3>, ((Trme <10>, Trfu <12>) <11>, (Naen <14>, Nasi <16>) <15>) <13>) <9>) <1>. Expansions and contractions are inferred relative to the reconstructed ancestral state at the parent node, not relative to the gene content of other extant species; a family may therefore be scored as expanded on a branch even where another genome retains more copies of it. Expanded, unchanged and contracted families sum to the 7245 orthogroups recovered across the nine genomes. “Genes gained” and “Genes lost” are the summed changes in gene number over all families in each category, and “Families lost” is the number of families whose copy number fell to zero on that branch. Expansions and contractions were scored as significant at a branch-wise Viterbi *p* < 0.01, and “Total significant changes” is the sum of the two preceding columns. The root node <1> is not reported, because CAFE assigns no change to it; node <9> shows no change because its stem branch is of essentially zero length, and no inference is drawn from that branch.

## Data Availability

The assembled genome sequences are publicly accessible through the National Center for Biotechnology Information (NCBI) under the BioProject accession PRJNA1480491. Raw sequencing reads have been archived in the Genome Sequence Archive (GSA), hosted by the China National Center for Bioinformation (CNCB), with the corresponding BioProject identifier PRJCA067427. All custom scripts developed for data analysis and figure generation are freely available via GitHub at https://github.com/huowenyanabace/T2Tfungi_custom_scripts.git (accessed on 1 June 2026).

## Supplementary Materials

The following supporting information can be downloaded at: https://www.mdpi.com/article/10.3390/jof12080602/s1, Figure S1: Per-chromosome Hi-C contact heatmaps for all 9 chromosomes of the *T. sanguinea* T2T genome assembly; Figure S2: k-mer frequency spectra and GenomeScope models for the *T. sanguinea* monosporic culture. Spectra were generated from quality-filtered short reads with Jellyfish (k = 21) and modelled with GenomeScope 2.0. (**a**) Single-haplotype model (*p* = 1): estimated haploid genome length 18.90–18.93 Mb, repeat content 0.85 Mb, model fit 89.1–91.3%. (**b**) Two-haplotype model (*p* = 2) with the initial k-mer coverage anchored at half the observed peak: estimated haploid genome length 20.92–20.96 Mb, repeat content 2.30 Mb, model fit 82.4–84.1%. The spectrum shows a single major peak, and both models converge on a haploid genome size close to that of the final assembly (21.44 Mb); the residual difference is accounted for mainly by the repeat fraction, which k-mer models are known to collapse; Table S1: Detailed Extension Statistics of *T. sanguinea*; Table S2: Genome assemblies andannotations of the nine Tremellales genomes analysed in this study; Table S3: Statistics of CAZyme gene numbers; Table S4: GO terms excluded prior to enrichment testing as not applicable to fungi; Table S5: Redundant GO terms collapsed into a single representative in Figure 5a; Table S6: Secondary metabolite biosynthetic gene clusters predicted by antiSMASH in six Tremellales genomes; Table S7: Coordinates and cluster types of all biosynthetic gene clusters predicted by antiSMASH; Table S8: Homology search for fungal pigment biosynthesis pathways across seven proteomes.
