# Supplementary material for: Telomere-to-Telomere Genome Assembly of Tremella sanguinea Corroborates Its Placement in Phaeotremella and Dates Its Divergence (Tremellales)
Source: J Fungi (Basel). 2026 Aug 13;12(8):602. doi: 10.3390/jof12080602 (PMC13514129; doi:10.3390/jof12080602)
Supplement: Supplementary file 1 [file jof-12-00602-s001.zip › Supplementary Figures.pdf]

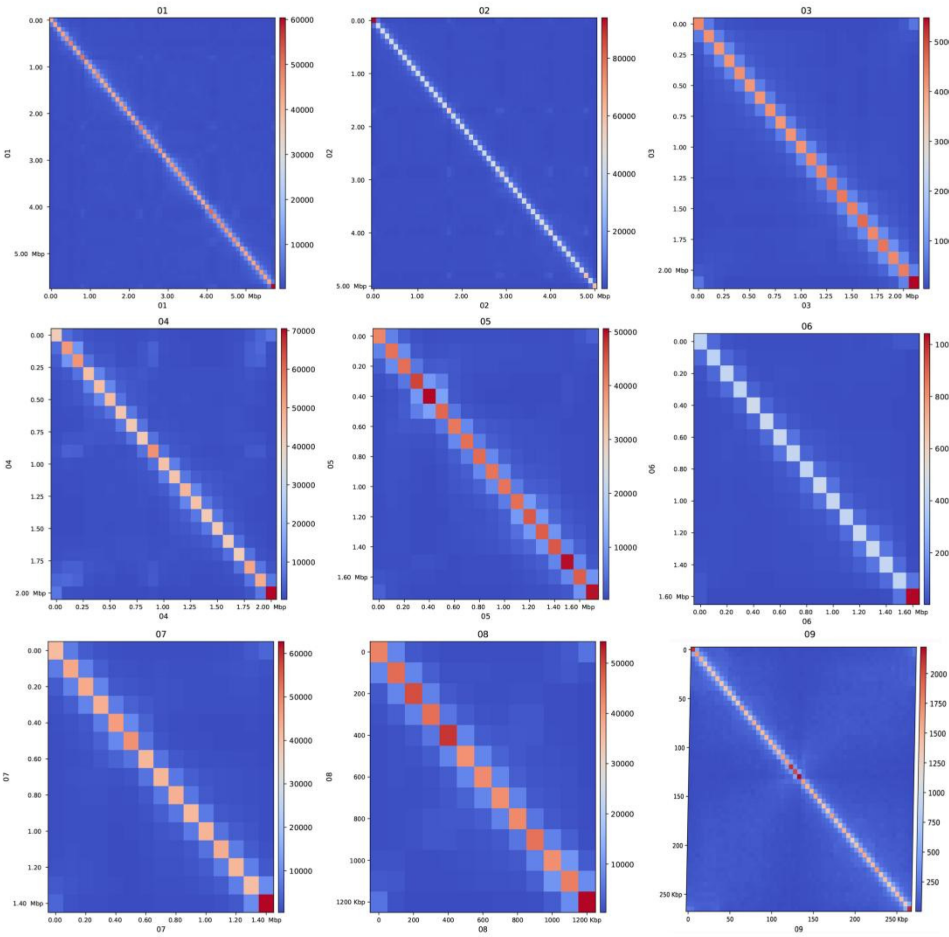

Figure S1. Per-chromosome Hi-C contact heatmaps for all 9 chromosomes of the *T. sanguinea* T2T genome assembly.

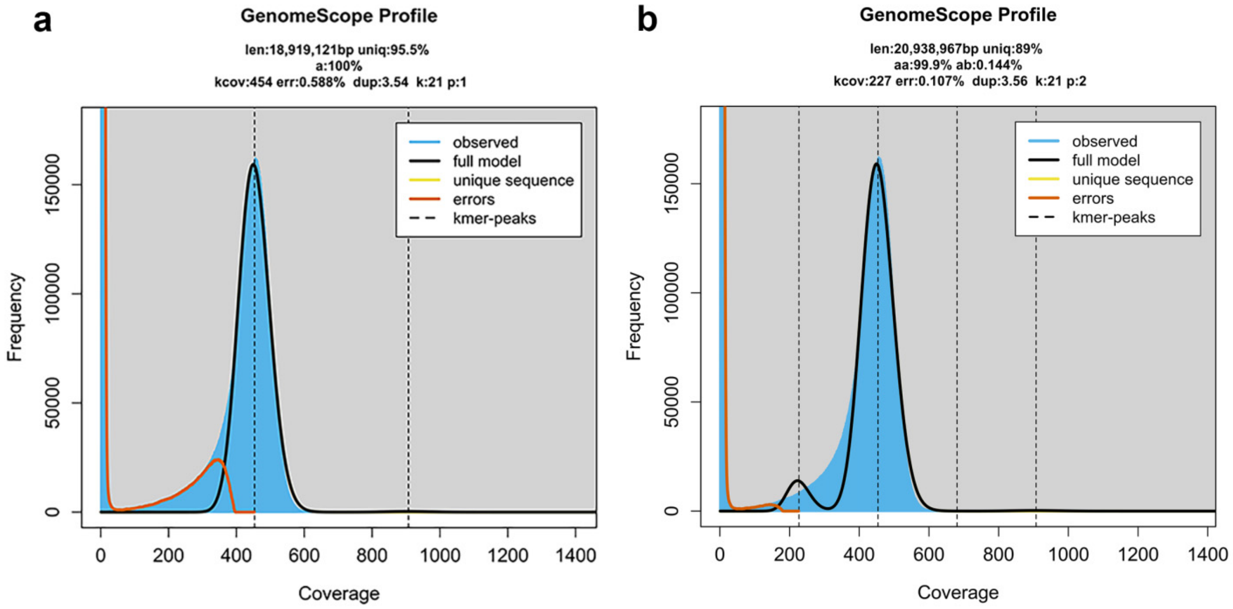

Figure S2. k-mer frequency spectra and GenomeScope models for the *T. sanguinea* monosporic culture. Spectra were generated from quality-filtered short reads with Jellyfish (k = 21) and modelled with GenomeScope 2.0. (**a**) Single-haplotype model (p = 1): estimated haploid genome length 18.90–18.93 Mb, repeat content 0.85 Mb, model fit 89.1–91.3 %. (**b**) Two-haplotype model (p = 2) with the initial k-mer coverage anchored at half the observed peak: estimated haploid genome length 20.92–20.96 Mb, repeat content 2.30 Mb, model fit 82.4–84.1 %. The spectrum shows a single major peak, and both models converge on a haploid genome size close to that of the final assembly (21.44 Mb); the residual difference is accounted for mainly by the repeat fraction, which k-mer models are known to collapse.
